# Analytical modeling of glucose biosensors based on carbon nanotubes

**DOI:** 10.1186/1556-276X-9-33

**Published:** 2014-01-15

**Authors:** Ali H Pourasl, Mohammad Taghi Ahmadi, Meisam Rahmani, Huei Chaeng Chin, Cheng Siong Lim, Razali Ismail, Michael Loong Peng Tan

**Affiliations:** 1 Faculty of Electrical Engineering, Universiti Teknologi Malaysia UTM, Skudai, Johor 81310, Malaysia; 2 Nanotechnology Research Center Nanoelectronic Group, Physics Department, Urmia University, Urmia 57147, Iran

**Keywords:** Carbon nanotube, SWCNT FET, Glucose detection, Biosensor, Analytical model, *I*-*V* characteristics, PBS, Glucose oxide

## Abstract

In recent years, carbon nanotubes have received widespread attention as promising carbon-based nanoelectronic devices. Due to their exceptional physical, chemical, and electrical properties, namely a high surface-to-volume ratio, their enhanced electron transfer properties, and their high thermal conductivity, carbon nanotubes can be used effectively as electrochemical sensors. The integration of carbon nanotubes with a functional group provides a good and solid support for the immobilization of enzymes. The determination of glucose levels using biosensors, particularly in the medical diagnostics and food industries, is gaining mass appeal. Glucose biosensors detect the glucose molecule by catalyzing glucose to gluconic acid and hydrogen peroxide in the presence of oxygen. This action provides high accuracy and a quick detection rate. In this paper, a single-wall carbon nanotube field-effect transistor biosensor for glucose detection is analytically modeled. In the proposed model, the glucose concentration is presented as a function of gate voltage. Subsequently, the proposed model is compared with existing experimental data. A good consensus between the model and the experimental data is reported. The simulated data demonstrate that the analytical model can be employed with an electrochemical glucose sensor to predict the behavior of the sensing mechanism in biosensors.

## Background

The advent of nanotechnology provides a new perspective for the development of nanosensors and nanoprobes with nanometer dimensions and is appropriate for biological and biomolecular measurements [1]. The use of tools capable of detecting and monitoring the biomolecular process can create enormous advances in the detection and treatment of diseases and thereby revolutionize cell biology and medical science [2]. A biosensor is an electronic device which has a biological probe and a transducer that is connected to a monitor. The demand for a wide variety of applications for a biosensor in industrial, environmental and biomedical diagnostics is dramatically increasing [1-3]. Biomedical applications, such as blood glucose detection, demand a great deal of research activities. Glucose oxide (GOx)-based enzyme sensors have been immensely used for the diagnosis and monitoring of blood glucose level because of the ability of GOx to identify glucose target molecules quickly and accurately [4-6]. Because of the constraints of other approaches, such as ultralow detection, large detection range, high cost, and knowledge complexity, the implementation of effective approaches using carbon-based materials is vital. Carbon nanotubes (CNTs) with superior electrical performance are essential in designing modern biosensors [7-10]. CNT-based biosensors have an economical production process, rapid response, high sensitivity, and good selectivity and are easily available in the market. Hence, a great deal of research has been conducted to study the performance of CNT-based field-effect transistor (FET) biosensors [11-14]. Due to their excellent mechanical stability, high conductivity, and antifouling properties, CNTs have been widely employed for GOx immobilization in biosensors [15]. Moreover, the CNT platform provides a more appropriate environment for immobilized GOx and therefore provides a quick shuttling of electrons with the surface of an electrode [15,16]. In sensor technology, analytical modeling based on experimental finding is still ongoing. This study proposes an analytical glucose biosensor model of single-wall carbon nanotube field-effect transistor (SWCNT FET) to predict the drain current versus drain voltage (*I*-*V*) performance. For the first time, the effects of glucose adsorption on CNT electrical properties, namely gate voltage, are studied and formulated versus a wide range of glucose concentration.

## Methods

### Sensing mechanism

**Figure 1:**
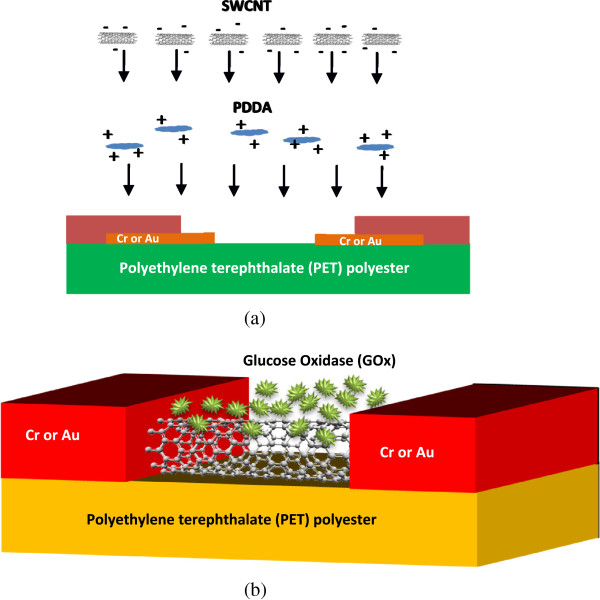
**Schematic fabrication process and a field-effect sensor. (a)** Schematic fabrication process of glucose sensor [24]. **(b)** Proposed combination of metal electrodes made of chromium or gold, a layer of GOx biomolecular assembly, and SWCNT channel in the form of FET.

In this section, the methods of immobilization will be described to explain the sensing mechanism of a biosensor. Immobilization is a process to integrate a biocatalyst with a matrix that it is not soluble in aqueous media. A wide variety of approaches can be applied for the immobilization of enzymes or cells on a variety of natural and synthetic supports. Both of the immobilization approach and support are dependent on the type of enzyme and substrate [17,18]. Enzymes are very instable and sensitive to their environment [19]. When no special precaution is required, some common approaches, such as deactivation on an adsorption and chemical or thermal inactivation, are adopted [19,20]. The important techniques that maintain the enzyme activity of immobilization are encapsulation, covalent immobilization, and site-specific mutagenesis [15,21]. Ultimately, the application of the new materials will generally affect the quality of the sensing mechanism. Because of the high surface area-to-volume ratio, CNTs demonstrate good device performance [22] when they are used as a semiconducting channel in biosensors [23]. The CNT application on glucose detection has been experimentally reported in [24] where GOx is utilized as an enzyme. The fabrication process of the SWCNT-based (1 to 2 nm in diameter, 50 μm in length) [25,26] electrochemical glucose biosensors using GOx [24] is depicted in Figure 1a,b. Polyelectrolytes, such as poly(diallyldimethylammonium chloride) (PDDA) and polystyrenesulfonate (PSS) are implemented [24]. Figure 1a shows the assembly of PDDA/SWCNT on polyethylene terephthalate (PET) polyester flexible substrate, and GOx biomolecular assembly is depicted in Figure 1b.

To produce stable negative charges, GOx is dissolved into a phosphate-buffered saline (PBS) with a concentration of 1 mg/mL. Phosphate monobasic (NaH_2_PO_4_) and dibasic (Na_2_HPO_4_) are employed as a standard pH buffer solution. The standard d-glucose solutions have been used in the glucose concentration test, and the results are shown in terms of drain current versus drain voltage (*I*-*V*) characteristics [24].

### Proposed model

**Figure 2:**
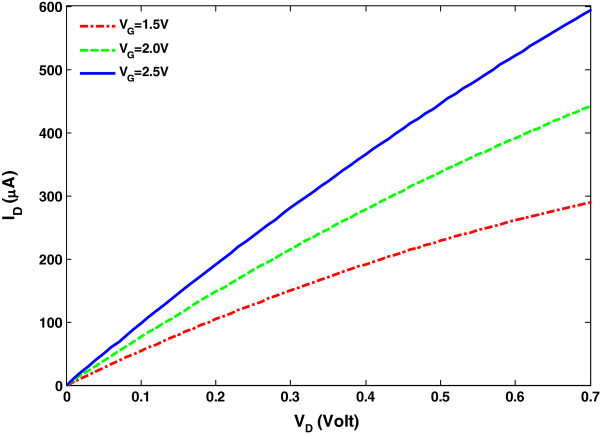
***I*-*V *** **characteristics of the SWCNT FET based on the proposed model for various gate voltages.**

Figure 1b shows the structure of the SWCNT FET with PET polyester as a back gate and chromium (Cr) or aurum (Au) as the source and drain, respectively. A SWCNT is employed as a channel to connect the source and drain. According to the proposed structure, two main modeling approaches in the carbon nanotube field-effect transistor (CNTFET) analytical modeling can be utilized. The first approach is derived from the charge-based framework, and the second modeling approach is a noncharge-based analytical model using the surface-potential-based analysis method. The charge-based carrier velocity model is implemented in this work. The drift velocity of carrier in the presence of an applied electric field [27] is given as

(1) $$v_{D}=\frac{\mathrm{\mu E}}{1+\frac{E}{E_{c}}}\;$$

where *μ* is the mobility of the carriers, *E* is the electric field, and *E*_*c*_ is the critical electric field under high applied bias. From Equation 1, the drain current as a function of gate voltage (*V*_G_) and drain voltage (*V*_D_) is obtained as

(2) $$I_{D}=\beta \frac{\left[2V_{\mathrm{GT}}V_{D}-V_{D}^{2}\right]}{1+\frac{V_{D}}{V_{c}}}$$

where *β* = *μC*_G_/(2*L*), *V*_GT_ = *V*_G_ - *V*_T_, and critical applied voltage as *V*_c_ = (*v*_sat_/*μ*)*L*, where *v*_sat_ is the saturation velocity, *V*_G_ is the gate to source voltage, *V*_T_ is the threshold voltage [28], *C*_G_ is the gate capacitance per unit length, and *L* is the effective channel length [29]. The unknown nature of the quantum emission is not considered in this calculation. Based on the geometry of CNTFET that is proposed in Figure 1b, the gate capacitance (*C*_G_) can be defined as

(3) $$C_{G}=\frac{C_{E}C_{Q}}{C_{E}+C_{Q}}$$

where *C*_E_ and *C*_Q_ are the electrostatic gate coupling capacitance of the gate oxide and the quantum capacitance of the gated SWCNT, respectively [30-33]. Figure 2 shows the *I*-*V* characteristics of a bare SWCNT FET for different gate voltages without any PBS and glucose concentration that is based on Equation 2.

The electrostatic gate coupling capacitance *C*_*E*_ for Figure 1b is given as

(4) $$C_{E}=\frac{2\pi \epsilon }{\ln\left(4H_{\mathrm{PET}}/d\right)}L$$

where *H*_PET_ is the PET polyester thickness, *d* is the diameter of CNT and *ϵ =* 3.3*ϵ*_*0*_ is the dielectric permittivity of PET. The existence of the quantum capacitance is due to the displacement of the electron wave function at the CNT insulator interface. *C*_Q_ relates to the electron Fermi velocity (*v*_F_) in the form of *C*_Q_ = 2e/*v*_F_ where *v*_F_ ≈ 10^6^ m/s [34]. Numerically, the quantum capacitance is 76.5 aF/μm and shows that both the electrostatic and quantum capacitances have a high impact on CNT characteristics [35,36]. At saturation velocity, the electric field is very severe at the early stage of current saturation at the drain end of the channel.

In this research, the effect of glucose concentration (*F*_g_) on the *I*-*V* characteristics of the CNTFET is studied. First, PBS should be added to the solution so that glucose is detectable and the change in *I*-*V* can be observed. The *I*-*V* change is due to the carrier concentration gradient of the injected carriers from the PBS to the channel and vice versa. The channel carrier concentration can be modeled in the function of gate voltage variations as

(5) $$V_{\mathrm{GS}1\left(\mathrm{with}\;\mathrm{PBS}\right)}=V_{\mathrm{GS}\left(\mathrm{without}\;\mathrm{PBS}\right)}+V_{\mathrm{PBS}}$$

where *V*_GS1(with PBS)_ is the gate voltage in the presence of PBS, *V*_PBS_ is the voltage due to the interaction of PBS with CNT in the solution, and *V*_GS(without PBS)_ indicates the gate voltage in a bare channel. The effect of PBS in the *I*-*V* characteristics is modeled as

(6) $$I_{D}=\frac{\left[2\left(V_{\mathrm{GS}\left(\mathrm{without}\;\mathrm{PBS}\right)}+V_{\mathrm{PBS}}-V_{T}\right)V_{D}-V_{D}^{2}\right]}{1+\frac{V_{D}}{V_{c}}}.$$

**Figure 3:**
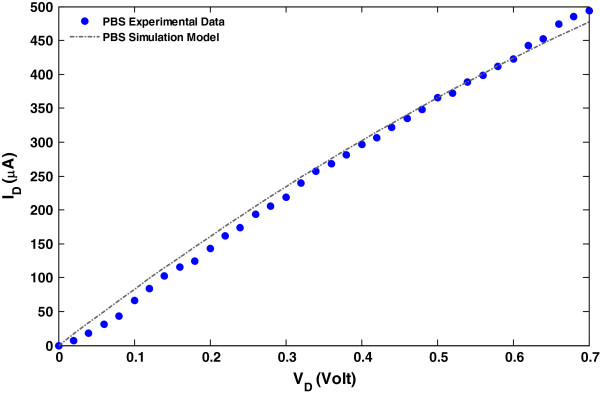
**Comparison of the *I*-*V *simulation output and the experimental data [**[24]**].** PBS concentration *F*_PBS_ = 1 mg/mL, *V*_GS(without PBS)_ = 1.5, and *V*_PBS_ = 0.6 V.

Before glucose and PBS is added, *V*_GS(without PBS)_ is set to be 1.5 V. The *V*_PBS_ is found to 0.6 V when the PBS concentration, *F*_PBS_ = 1 mg/mL, is added into the solution. Using Equations 5 and 6, the presented model provides a good consensus between the model and the experimental data as shown in Figure 3.

In the glucose sensing mechanism reported in [24], β-d-glucose oxidizes to d-glucono-δ-lactone and hydrogen peroxide (H_2_O_2_) as a result of the catalyst reaction of GOx. The hydrolyzation of d-glucose-δ-lactone and the electrooxidation of H_2_O_2_ under an applied gate voltage produce two hydrogen ions and two electrons which contribute to the additional carrier concentration in the SWCNT channel. On the whole, the glucose sensing mechanism can be summarized as follows:

(7) $$\begin{array}{ll} \beta ‒D‒\text{glucose} & +O_{2}\;\mathrm{with}\;\mathrm{GOx}\;\mathrm{enzyme}\to D‒\mathrm{glucono}‒\delta ‒\text{lactone}\\ & +H_{2}O_{2} \end{array}$$

(8) $$D‒\mathrm{glucono}‒\delta ‒\mathrm{lactone}+H_{2}O\;\to \;D‒{\mathrm{gluconate}}^{-}+H^{+}$$

(9) $$H_{2}O_{2}\;\to \;O_{2}+2H^{+}+2e$$

The variation of the proximal ionic deposition and the direct electron transfer to the electrode surface modify the electrical conductance of the SWCNT. The direct electron transfer leads to a variation of the drain current in the SWCNT FET. Therefore, Equation 10 that incorporates the gate voltage change due to the additional electrons from the glucose interaction with PBS is given as

(10) $$\begin{array}{ll} V_{\mathrm{GS}2\left(\mathrm{with}\;\mathrm{PBS}\;\mathrm{and}\;\mathrm{glucose}\;\mathrm{detection}\right)}= & V_{\mathrm{GS}\left(\mathrm{without}\;\mathrm{PBS}\right)}\\ & +V_{\mathrm{PBS}}+V_{\mathrm{Glucose}} \end{array}$$

By incorporating Equation 10, Equation 6 then becomes

(11) $$I_{D}=\frac{\left[2\left(V_{\mathrm{GS}\left(\mathrm{without}\;\mathrm{PBS}\right)}+V_{\mathrm{PBS}}+V_{\mathrm{Glucose}}-V_{T}\right)V_{D}-V_{D}^{2}\right]}{1+\frac{V_{D}}{V_{c}}}.$$

*V*_Glucose_ is the glucose-based controlling parameters that highlight the effects of glucose concentration against gate voltages. In the proposed model, Equation 12 is obtained by analyzing the rise *I*_D_ with gate voltages versus glucose concentration. Based on the iteration method demonstrated in [37], the concentration control parameter as a function of glucose concentration in a piecewise exponential model is expressed as

(12) $$V_{\mathrm{Glucose}}\left(F_{g}\right)=\;\left\{\begin{array}{ll} 0\;V & \mathrm{if}\;F_{g}=0\;\mathrm{mM}\\ 1.42\;V-\exp\left(-0.1\times F_{g}\right) & \mathrm{if}\;F_{g}>0\;\mathrm{mM} \end{array}\right)\;.$$

In other words, the *I*-*V* characteristics of the biosensor can also be controlled by changing the glucose concentration. To evaluate the proposed model, the drain voltage is varied from 0 to 0.7 V, which is similar to the measurement work, and *F*_g_ is changed in the range of 2 to 50 mM [24].

## Results and discussion

### Glucose sensing and accuracy of sensor model

**Figure 4:**
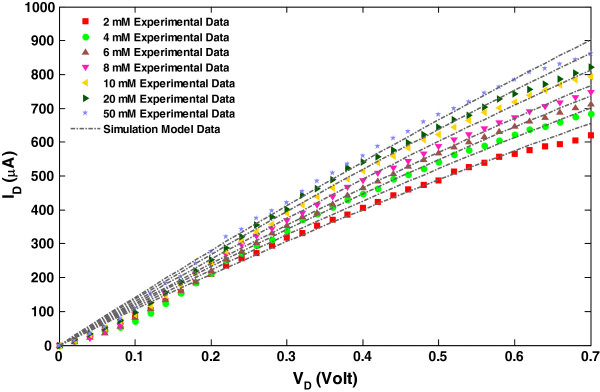
***I*-*V *comparison of the simulated output and measured data [**[24]**] for various glucose concentrations.***F*_*g*_ = 2, 4, 6, 8, 10, 20, and 50 mM. The other parameters used in the simulation data are *V*_GS(without PBS)_ = 1.5 V and *V*_PBS_ = 0.6 V.

By increasing the glucose concentration in multiple steps from 2 to 50 mM, a fairly good consensus between our simulation model and experimental data particularly in the linear region is illustrated in Figure 4. The results show the accuracy of our predictive model against the measurement data of the glucose biosensor for various glucose concentrations up to 50 mM. It is observed that the current in the CNTFET increases exponentially with glucose concentration.

From Figure 4, the glucose sensor model shows a sensitivity of 18.75 A/mM on a linear range of 2 to 10 mM at *V*_D_ = 0.7 V. The high sensitivity is due to the additional electron per glucose molecule from the oxidation of H_2_O_2_, and the high quality of polymer substrate that are able to sustain immobilized GOx [24]. It is shown that by increasing the concentration of glucose, the current in CNTFET increases. It is also evident that gate voltage increases with higher glucose concentrations. Table 1 shows the relative difference in drain current values in terms of the average root mean square (RMS) errors (absolute and normalized) between the simulated and measured data when the glucose is varied from 2 to 50 mM. The normalized RMS errors are given by the absolute RMS divided by the mean of actual data. It also revealed that the corresponding average RMS errors do not exceed 13%. The discrepancy between simulation and experimental data is due to the onset of saturation effects of the drain current at higher gate voltages and glucose concentration where enzyme reactions are limited.

**Table 1:** Average RMS errors (absolute and normalized) in drain current comparison to the simulated and measured data for various glucose concentration

**Glucose (mM)**	**Absolute RMS errors**	**Normalized RMS errors (%)**
0 (with PBS)	19.24	5.66
2	57.55	12.22
4	49.05	9.75
6	59.47	11.23
8	53.99	9.80
10	55.60	9.53
20	69.18	11.17
50	75.07	11.60

## Conclusions

The CNTs as carbon allotropes illustrate the amazing mechanical, chemical, and electrical properties that are preferable for use in biosensors. In this paper, the analytical modeling of SWCNT FET-based biosensors for glucose detection is performed to predict sensor performance. To validate the proposed model, a comparative study between the model and the experimental data is prepared, and good consensus is observed. The current of the biosensor is a function of glucose concentration and therefore can be utilized for a wide process variation such as length and diameter of nanotube, capacitance of PET polymer, and PBS voltage. The glucose sensing parameters with gate voltages are also defined in exponential piecewise function. Based on a good consensus between the analytical model and the measured data, the predictive model can provide a fairly accurate simulation based on the change in glucose concentration.

## Competing interests

The authors declare that they have no competing interests.

## Authors’ contributions

AHP designed and performed the device modeling and simulation work, analyzed the data, and drafted the manuscript. MLPT, MTA, and RI supervised the research work, and MR assisted with the carbon nanotube device modeling. MLPT proofread the manuscript, and HCC improved the quality of the figures through MATLAB simulation. MLPT and CSL provided the funding for the research. All authors read and approved the final manuscript.

## Authors’ information

AHP received his B.S. degree in Electronic Engineering from the Islamic Azad University of Bonab, Iran in 2011. At the moment, he is pursuing his Master’s degree in Eng. (Electronic and Telecommunication) from Universiti Teknologi (UTM), Malaysia. He is currently a member of the Computational Nanoelectronics (CoNE) Research Group in UTM. His current research interests are in biosensors based on nanomaterials and nanodevices.

MTA is a tenured assistant professor of nanoelectronics at the Nanotechnology Research Center at Urmia University. He received his Ph.D. degree in Electrical Engineering from Universiti Teknologi Malaysia in 2010. His research interests are in the simulation, modeling, and characterization of nonclassical nanostructure devices which include sensors and transistors.

MR received his Ph.D. degree in Electrical Engineering from UTM in 2013. He joined the Computational Nanoelectronics (CoNE) Research Group in 2009. He has published over 20 peer-reviewed papers in reputed international journals and conferences. His main research interests are in carbon-based nanoelectronics.

HCC was born in Bukit Mertajam, Penang, Malaysia, in 1989. She received her B. Eng. (electrical-electronics) from Universiti Teknologi Malaysia (UTM) in 2013. During her practical training, she underwent an internship at Intel Penang Design Centre, Penang, Malaysia. She is currently pursuing her Master’s degree at the same university.

CSL received his B. Eng. degree in Electrical Engineering (first class honors), M. Eng degree (Electrical), and Ph.D. degree from Universiti Teknologi Malaysia (UTM), in 1999, 2004, and 2011, respectively. He is a senior lecturer at UTM, a faculty member of the Department of Control and Mechatronic Engineering, and a research member of Process Tomography Research Group & Instrumentation (PROTOM-i), Faculty of Electrical Engineering. His research interests are in embedded system, emergency medical services, telerobotics and multi-agent system.

RI received his B.Sc. and M.Sc. degrees in Electrical and Electronic Engineering from the University of Nottingham, Nottingham, UK in 1980 and 1983, respectively, and his Ph.D. degree from Cambridge University, Cambridge, UK in 1989. In 1984, he joined the Faculty of Electrical Engineering, Universiti Teknologi Malaysia as a lecturer in Electrical and Electronic Engineering. He has held various faculty positions including head of the department and chief editor of the university journal. RI has worked for more than 20 years in this research area and has published various articles on the subject. His current research interest is in the emerging area of nanoelectronic devices focusing on the use of carbon-based materials and novel device structure. He is presently with the Universiti Teknologi Malaysia as a professor and head of the Computational Nanoelectronics (CoNE) Research Group. RI is a member of the IEEE Electron Devices Society (EDS).

MLPT was born in Bukit Mertajam, Penang, Malaysia, in 1981. He received his B. Eng. (Electrical-Telecommunications) and M. Eng. (Electrical) degrees from Universiti Teknologi Malaysia (UTM), Skudai, Malaysia, in 2003 and 2006, respectively. He conducted his postgraduate research in nanoscale MOSFET modeling at the Intel Penang Design Center, Penang, Malaysia. He recently obtained his Ph.D. degree in 2011 at the University of Cambridge, Cambridge, UK. He is a senior lecturer at UTM, a faculty member of the Department of Electronic and Computer Engineering and a research member of the CoNE Research Group, Faculty of Electrical Engineering. His present research interests are in device modeling and circuit simulation of carbon nanotube, graphene nanoribbon, and MOSFET. MLPT is a registered graduate engineer of BEM, IEEE member, MIET member, graduate member of IEM (GRAD IEM), MySET, Johor Bahru Toastmasters International Club, and alumnus of Queens’ College Cambridge.
